# Comparison of pharmacological and nonpharmacological treatment strategies in promotion of infertility self-efficacy scale in infertile women: A randomized controlled trial

**Published:** 2013-06

**Authors:** Hajar Pasha, Mahbobeh Faramarzi, Seddigheh Esmailzadeh, Farzan Kheirkhah, Hajar Salmalian

**Affiliations:** 1 *Fatemeh Zahra Infertility and Reproductive Health Research Center, Babol University of Medical Sciences, Babol, Iran.*; 2 *Department of Psychiatry, Faculty of Medicine, Babol University of Medical Sciences, Babol, Iran.*

**Keywords:** *Infertility*, *Self-efficacy*, *Cognitive behavioral therapy*, *Psychotherapy*, *Drug therapy*

## Abstract

**Background:** The infertility is associated with psychological consequence including depression, and lack of self-efficacy.

**Objective:** The aim of this study was to compare the pharmacological and no pharmacological strategies in promotion of self-efficacy of infertile women.

**Materials and Methods:** A randomized controlled clinical trial was conducted on 89 infertile women who were recruited from Fatemeh Zahra Infertility and Reproductive Health Research Center and were randomized into three groups; cognitive behavioral therapy (CBT), antidepressant therapy with flouxetine 20 mg daily for 3 month, and a control group. All participants completed Infertility Self-efficacy Inventory (ISE) and the Beck Depression Inventory (BDI) at the beginning and end of the study.

**Results:** The means ISE scores among the CBT, fluoxetine, and control groups at the beginning and end of the study were 6.1±1.6 vs. 7.2±0.9, 6.4±1.4 vs. 6.9±1.3 and 6.1±1.1 vs. 5.9±1.4 respectively. Both CBT and fluoxetine increased the mean of ISE scores more than control group after intervention (p<0.0001, p=0.033; respectively), but increase in the CBT group was significantly greater than flouxetine group. Finally, there was evidence of high infertility self-efficacy for women exposed to the intervention compared with those in the control group. Also, there was an improvement in depression. Both fluoxetine and CBT decreased significantly the mean of BDI scores more than the control group; decrease in the CBT group was significantly more than that in the fluoxetine group.

**Conclusion:** CBT can serve as an effective psychosocial intervention for promoting self-efficacy of infertile women.

**Registration ID in IRCT:** IRCT2012061710048N1

## Introduction

Infertility is a unique medical challenge (1). The inability to conceive children are experienced as heartrending condition that can cause stress in infertile couples (1, 2). Infertility and psychological difficulties are interrelated with each other. Some know infertility as a psychosomatic problem but others, who have more supporters, know psychological stress as the result of infertility. 

According to this hypothesis, the experience of infertility affects the infertile couple with deep emotional tensions which is the fixed source of psychological and social dreads (3). Various studies admit a rise in anxiety, depression and low self-confidence about 36-50.6% (2, 4-8). How to cope with infertility depends on the intensity of stress; ways of managing in spite of difficulty, social support, personality characteristic, and self-confidence (1, 2).

Several studies showed that self-efficacy have the important role in health promotion and outcome (9, 10). Self-efficacy is a person’s belief in his or her capacity to succeed in a particular situation. Bandura reported that these beliefs can effect on how person think, act, and feel (10). The studies showed that self-efficacy can influence biological indicators in reproductive health. Women with high self-efficacy have more positive emotional state, urging with infertility treatment and in this point psychological intervention may be helpful (11, 12). With attention to infertility crisis, cognitive policy can be an important main component in managing infertility (13, 14). 

Cousineau and Bastani believed that although drug therapy is useful as a short-term treatment, but psychological interventions is very important for reconstructing body satisfaction in infertile women (1, 15). The similar studies showed that cognitive behavior therapy (CBT) was superior to fluoxetine in the resolution depression, anxiety of infertile women and also psychotherapy is a reliable alternative to pharmacotherapy to promote the mental health of infertile women )^4^, ^16^). 

As, few studies have examined efficacy of psychotherapy and pharmacotherapy on infertility self-efficacy and there are no published data about efficacy of CBT versus fluoxetine intervention on infertility self-efficacy in Iranian infertile women set, therefore this study conduct to compare the effectiveness of CBT with fluoxetine on promotion of infertility self-efficacy in a sample of Iranian infertile women. 

## Materials and methods

A randomized controlled clinical trial was conducted in Fatemeh Zahra Infertility and Reproductive Health Research Center of the Babol University of Medical Sciences from September 2006 to June 200 7. The trial was registered at the Iranian registry of clinical trials and was approved by Ethical Committee of Babol University of Medical Sciences. Also, Deputy of Research of Babol University of Medical Sciences was financial support of the study. It should be noted that results of this articles is part of an extend project that implemented in 2007. Comparison of pharmacotherapy and psychotherapy in improvement of depression, anxiety, and general health reported in previous publications (4, 16). This article focuses on comparison of two types treatment in increasing of infertility self- efficacy.

After coordination and receiving justification from Infertility Council, infertile women who had dossier there, were recruited for this study. Women with these characteristics were invited to this study: age of fewer than 45 years, more than five years of education, more than two years of infertility, having at least one IVF, were not undergoing fertility treatment until 3 months afterward, were not currently practicing any relaxation techniques, were not participating in any support group, were not currently taking any psychotherapy, and were not currently undergoing any assisted reproductive therapy. Five midwives of the center conducted structured telephone invitations with potential participants. 

**Figure 1:**
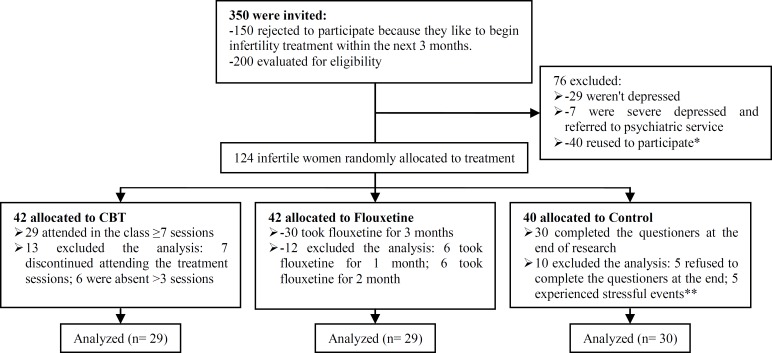
Flow diagram of participants through each stage of randomized, controlled trial.

Of 350 invitations, 200 patients with informed consent accepted to enter the study and were referred to the center. Subsequent to completing the demographic questionnaire and the Beck Depression Inventory (BDI), a psychologist conducted a face-to-face interview. Women who met one of the following conditions were excluded from the study: a score ≤9 or >47 on the BDI, or meeting the criteria for clinical severe depression on the clinical interview. Thus, only women with minimal, mild, and moderate depression (Beck score 10-47) were included in the study. Figure 1 shows the flow diagram of participants through each stage of randomized, controlled trial. Finally, 89 participants remained until the end of the study that they were put in 3 groups through a randomized, controlled trail (29 CBT, 30 fluoxetine, and 30 controls). The block randomization was by a paper list (random numbers supplied from 1-89 by the trial statistician) prepared by an investigator with no clinical involvement in the trial.

Participants in the CBT group were engaged in a 10-week, two-hour group cognitive behavior therapy program. Progressive muscle relaxation was added to sessions 5-10. Groups consisted of 8-12 members and the therapist was an expert psychologist who trained for the CBT program. Therapy was conducted at the Psychiatry Department of the Babol University of Medical Sciences. The first three sessions provided patients with a general orientation to cognitive therapy and the causes of infertility. A gynecologist participated in the first three sessions for 30 minutes and explained the cause of infertility for each patient. The following three sessions (sessions 4-6) included the identification and challenging of core dysfunctional or irrational beliefs that underlie automatic negative thoughts about the infertility. 

Finally, sessions 7-10 taught participants varying techniques (e.g. countering, and self-reward) for maintaining the change of their dysfunctional beliefs about infertility. In addition to the above program, sessions 5-10 taught participants progressive muscle relaxation (17, 18). in a group setting For home practicing, subjects read a relaxation book and listened to a 20-minute pre-recorded CD two times daily over a period of 5 weeks. Cognitive sessions were conducted based on Beck Depression Inventory and tension releasing sessions were conducted based on Relaxation Inventory of Jacobson (19, 20). 

The pharmacotherapy group took a capsule of fluoxetine (20 mg, Darou pakhsh, Iran) daily for 90 days. After the interview, the subjects took 30 capsules from the midwives of the center and return monthly to get their drug. The control group didn’t undergo any drug or psychological intervention. They completed questionnaires at the beginning of the study and 3 months after the interview. In CBT group during these 3 months, those subjects who had therapy interventions in vitro fertilization, anti-depression or psychological drug) or those who had stressful events such as family death were excluded. 

All participants completed the BDI and Self- Efficacy Inventory (SEI) at the beginning and end of the study. BDI and SEI used were reliable instruments to assess perceived depression and also self-efficacy for coping with a diagnosis and treatment for infertility (1). This instrument contains 16 item and respondent rate each item based on a likert scale such as ''not at all confident'' to ''very confident'. The infertile women were asked to rate to what fell confidence when receiving treatment for infertility, using a 9-point response scale ranging from 1= “Not at all confident” to 9= “Totally confident". 

The same study showed that the ISE is a relatively homogeneous scale and inter item correlations for self- efficacy item was sufficient inter relationship. The Cronbachs estimate of internal consistency for 16 item ISE scale was 0.94. The item total correlations ranged from 0.59 to 0.86 and test -retest reliability 91% (1). In this sample, the internal consistency reliability of ISE was 0.80.There was a meaningful negative correlation (p<0.01) between Self-efficacy Inventory and Beck Depression Inventory (-0.251). The mean score ISE of infertile women was obtained after calculating the scores and then dividing received scores on the number of questions. The least mean score based on Likret index was 1“not at all confident” and the highest one was 9" Totally confident”.

**Statistical analysis**

Data were compared by Paired t-tests, ANOVA, x^2^, and Post-Hoc (Tukey) test. Significance was denoted by p<0.05. Statistical analysis were performed SPSS 17.

*Some of 40 depressed women who refused to enter the study denied their mood disorders, and some of them believed that they could find relief from depression without medication intervention. Some of them worried if they began their treatment course, their family would mark them as mental patients.

**As stressful events such as family death, heavy accidents, pregnancy, etc. affected the stress infertility or depression tests, these women excluded the analysis.

* Cognitive behavioral therapy.

** ANOVA was performed to compare the means of groups.

## Results

The study results confirmed that there were no statistically significant differences among the three groups in age, educational level, economical status, and the duration of infertility. The majority of women's job in CBT, fluoxetine, and the control group was housekeeper (92.6%, 93.3%, and 100%).The demographic characteristics of the study sample are summarized in table I. 

**Figure 2:**
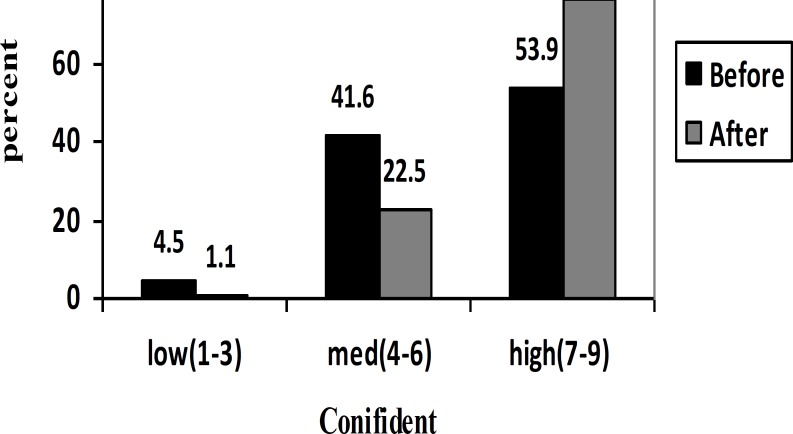
levels of infertility self-efficacy in infertile women (before and after intervention)

The means ISE scores among the CBT, fluoxetine, and control groups at the beginning and end of the study were changed (Table II). In total, the percentage of high confidence from infertility self-efficacy increased after intervention (Figure 2). There was evidence of high infertility self-efficacy for women exposed to the intervention compared with those in the control group, especially CBT group (Table III). The results had showed an improvement in infertility self-efficacy after intervention. Although the three groups did not have significantly differences between means ISE scores with ANOVA analysis at the beginning of the research, the difference was significant at the end of study (p<0.0001). 

Also in both CBT and fluoxetine groups, the mean of ISE scores increased significantly more than control group (p<0.0001, p=0.004); the Tukey test showed that increase in the CBT group was significantly greater than flouxetine group. Paired t-test approved that the difference of means was significant (Table III). The mean of Beck scores in CBT group and fluoxetine decreased after interventions (p<0.0001), but there was no significant difference in control group. Decrease in the CBT group was significantly more than that in the fluoxetine group.

**Table I:** Demographic and social characteristics by group

**Criteria**	**CBTa mean (SD)**	**Fluoxetine mean (SD)**	**Control mean (SD)**	**Fb**	**p-value**
Age (year)	28.3 (3.8)	29.8 (5.3)	28.4 (5.3)	0.8	0.44
Husband age (year)	33.4(4.2)	32.3(4.4)	33.9(6.3)	0.8	0.46
Education (year)	9.2 (2.4)	9.4 (4.2)	9.8 (3.9)	0.4	0.20
Husband education (year)	11.2 (3.4)	9.8 (4.6)	9.8 (3.1)	1.3	0.27
Duration of infertility (year)	5.4 (3.9)	6.3 (3.4)	5.7 (4.5)	0.4	0.7

a) Cognitive behavior therapy.

b) ANOVA was performed to compare the means of groups

**Table II:** Comparison the mean of ISE scores in each group (beginning and end of the study)

**Groups**	**At beginning [mean (SD)]**	**At ending [mean (SD)]**	**t a**	**p-value**
CBTb	6.1 (1.6)	7.2 (0.9)	-4.5	<0.0001
Fluoxetine	6.4 (1.4)	6.9 (1.3)	-2.2	0.033
Control	6.1 (1.1)	5.9 (1.4)	1.6	0.121

a) Pair t-test was used to compare the mean of scores, before and after interventions.

b) Cognitive behavioral therapy.

**Table III:** Levels of infertility self-efficacy in groups (beginning and ending of intervention)

**Groups**	**CBT**		**Fluoxetine**		**Control**	
**At beginning N (%)**	**At ending ** **N (%)**	**At beginning N (%)**	**At ending ** **N (%)**	**At beginning N (%)**	**At ending ** **N (%)**
Low (1-3)	2 (6.9)	0 (0)	2 (6.7)	0 (0)	0 (0)	1 (3.3)
Med (3-6)	13 (44.8)	2 (6.9)	8 (26.7)	4 (13.3)	16 (53.3)	14 (46.7)
High (7-9)	14 (48.3)	27 (93.1)	20 (66.7)	26 (86.7)	14 (46.7)	15 (50)

## Discussion

The aim of this project was to compare self-report measure of infertility self-efficacy in 3 groups of infertile women. The results showed that almost half of the infertile women weren’t with high self-efficacy. The mean of ISE Score was 6.18±1.39. Studies had shown that psychological factors can be involved in causing infertility and also infertility can be associated with psychological consequences such as low self-esteem and low self-efficacy (21). Cousineau *et al* showed that the mean ISE score was 5.8±1.6 and also women tended to have significantly lower self-efficacy scores on the ISE than men (6.7±1.5) (1). Principally, infertility causes feelings of helplessness, feelings of worthlessness and incompetence (2). Various studies admit a low self-confidence (6). 

Fisher believed that Infertility is as a crisis in life and if a woman is considered infertile, her own body image is changed (22). Results of this project demonstrated that CBT promoted self- efficacy of infertile women. CBT had a significant increase in ISE scores. Some other studies support this finding (1, 3, 12-14, 23, 24). Psychologist intervention increased self-esteem and self-acceptance (25). Principally, infertility is a life crisis and infertile women require coping with diagnosis and treatment of infertility (1, 26). Bandura *et al* believed that infertility stressful situation can influence on personal control, therefore an infertile woman must have the confidence and skills required to perform treatment process and at this point psychological intervention may be helpful (12, 27). 

The same studies showed that psychological intervention based on self-efficacy theory can have a positive impact on reducing anxiety and perceived stress and relaxation education can increase self-efficacy score in intervention group (3, 15, 24). Cognitive-behavioral techniques are useful and can greatly help to control symptoms (28). In the same study it was showed that psychological interventions is very important for reconstructing body satisfaction in infertile women (1). Andrew *et al* in a meta-analysis study showed support the efficacy of cognitive behavioral treatment for many psychiatric disorders (29). 

Matsunaga *et al* believed that CBT had a positive effect on social function in patients who showing medication treatment resistant depression (30). Therefore, with attention to infertility crisis, cognitive policy can be an important main component in managing infertility (13, 14). Also, it is one of non-drug method, cheap and harmless. It suggested reducing psychological problem (31). The gathering data suggested that CBT was better or greater than fluoxetine to promote or increase infertility self-efficacy.The studies had showed that fluoxetine is an antidepressant and its efficacy was also demonstrated in the treatment of depression, and alleviating anxiety (4, 16, 32). Therefore, it is expected that with improvement in depression and anxiety of infertile women have changed their body image and their self-efficacy will be enhanced. 

But it must be acknowledged that fluoxetine have side effects such as confusion, drowsiness, unusual weakness or fatigue, headache, dry mouth, nausea, cramps, abdominal pain, although it can depended to dose of drug (33). Bastani *et al* suggested that although drug therapy is useful as a short-term treatment, but long-term impact has not been approved and side effects of medications may have other problems (15). The finding of this study showed that the resolution of depression in the three groups was: CBT 79.3%, fluoxetine group 50%, and control 10%.

Although both fluoxetine and CBT decreased significantly the mean of BDI scores more than that of the control group. Both CBT and fluoxetine were effective on decreasing the depression and were superior to control group. Some similar studies know CBT as the effective therapy for depression (34). There are results showing that psychological interventions could positively help decrease the depression (35, 36). The different controlled studies evaluated the effect of CBT on depression and they showed that CBT was not only effective method on treatment of depression but also was superior to pharmacotherapy. Also, sometimes the CBT plus fluoxetine was superior to each of them individually (37).

There were a number of limitations in implementation of the current study. The first limitation was that 40 mild to moderate depressed women did not agree to enter treatment protocol. The second limitation was the number of dropouts from experimental and control groups. Fortunately, as demographic characteristics of women before dropouts were the same as after dropouts, it is found that the number of dropouts might have not biased the data in favor of the interventions. Third limitation was cultured band difference in north of IRAN that was a variable response to CBT or compliance for treatment. Forth limitation referred to the nature of two type of treatment. 

CBT group received more treatment than drug group and the positive results obtained may be due to more treatment (e.g., more contact, etc.) rather than anything specific about CBT.

## Conclusion

Interventions improved infertility self-efficacy level. Both CBT and Fluoxetine can facilitate the process of promoting self-efficacy in infertile women, but CBT intervention have the more beneficial effects in increased infertility self-efficacy. Therefore, CBT Method may serve as an effective, original psychosocial intervention and may be a cost effective resource for fertility practices and it is a reliable alternative of pharmacotherapy to increase infertility self-efficacy. Finally, the results of this study represent an serious step toward development of infertility self-efficacy by psychosocial intervention. 
